# A Case Report of Nonvasculitic Autoimmune Inflammatory Meningoencephalitis with Sensory Ganglionopathy: A Rare Presentation of Sjögren Syndrome

**DOI:** 10.1155/2017/5696512

**Published:** 2017-01-15

**Authors:** João Peres, Simão Cruz, Rita Oliveira, Luís Santos, Ana Valverde

**Affiliations:** ^1^Neurology Department, Hospital Professor Doutor Fernando Fonseca, Amadora, Portugal; ^2^Anatomic Pathology Department, Hospital Professor Doutor Fernando Fonseca, Amadora, Portugal; ^3^Neurology Department, Hospital de Egas Moniz-Centro Hospitalar Lisboa Oeste, Lisbon, Portugal

## Abstract

A 68-year-old Caucasian female was admitted to the emergency department with a progressive history of behavioural symptoms and anxiety followed by visual and auditory hallucinations, forgetfulness, and impaired gait in the previous 3 months. On examination she was psychotic and had a postural and rest tremor of the upper limbs, cogwheel rigidity of the four limbs, retropulsion on standing position, and inability to walk. During the following 2 weeks she developed xerostomia and unilateral parotiditis that improved with steroids. A simultaneous improvement of the cognitive abilities allowed for the detection of sensory ataxia of the lower limbs. Sensory ganglionopathy was then detected with electrophysiological studies. A diagnosis of Sjögren syndrome was suspected and confirmed by salivary gland scintigraphy, Schirmer's test, and submaxillary gland biopsy. We report a case of Sjögren syndrome associated with central and peripheral nervous system involvement, without* sicca* symptoms preceding the neurological clinical picture. The coexistence of ganglionopathy and a favourable response to immunosuppression are key features that can lead to the correct diagnosis in cases with atypical CNS symptoms, mimicking a rapidly progressive dementia.

## 1. Introduction

Sjögren syndrome is a common autoimmune disease that can be either primary or secondary to other autoimmune diseases. Its diagnosis is often difficult because of the wide clinical heterogeneity. Its histopathologic hallmark is the periductal lymphocytic infiltrate with destruction of salivary and lacrimal glands resulting in loss of their function, which leads to* sicca* symptoms. Parallel mononuclear infiltrate or vasculitic lesions may affect other organs and systems. Nervous system is one of the most common extraglandular involved sites, and it is reported to precede the* sicca* symptoms in a proportion of patients [1]. Neurological manifestations can involve both peripheral and central nervous systems. In this case the authors present a case of Sjögren syndrome which manifested as rapidly progressive dementia with impaired gait, in the absence of previous* sicca* symptoms.

## 2. Case Report

A 68-year-old Caucasian woman was taken by her son to the emergency department with a 3-month progressive history of anxiety followed by complex visual and auditory hallucinations, forgetfulness, and impaired gait in the previous 2 weeks. She had a history of Hashimoto thyroiditis for over 10 years and was on thyroid hormone replacement therapy with levothyroxine, without other relevant personal or family history. General physical examination was unremarkable. On admission she was alert, agitated, with frightened appearance and looking around with suspiciousness. She showed space and time disorientation, impaired attention, and incoherent speech, verbalizing nonsystematized delusional thoughts of persecutory type. Cranial nerve examination was normal. She had a postural and rest upper limbs tremor, cogwheel rigidity of the four limbs, brisk deep tendon reflexes, and a tendency to fall backwards on standing position and was unable to walk.

Diagnostic workup was performed with an initial clinical hypothesis of a rapidly progressive dementia. CSF examination revealed no cells and an increased protein content (74 mg/dL of the IgG type) with absent oligoclonal bands and with normal serum immunoglobulin concentration. Test for 14-3-3 protein was negative. EEG detected a generalized slowing of background activity, without epileptiform activity. Brain MRI showed only mild diffuse cortical atrophy.

**Figure 1:**
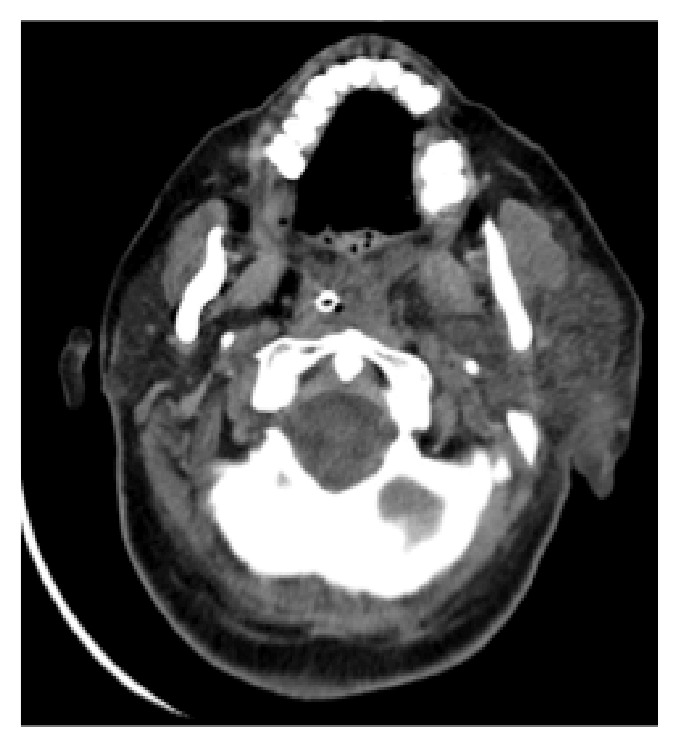
Head CT demonstrating left parotiditis.

On day 7 after admission, the patient developed xerostomia and unilateral left parotiditis (Figure 1) that remitted under oral prednisolone 40 mg once a day. This treatment also made a marked improvement in cognitive and psychiatric symptoms over the following days. Neurological reassessment on the 14th day showed weak deep tendon reflexes and decreased vibration and position senses on lower limbs, with sensory ataxia, Romberg sign, and wide-based gait. Nerve conduction studies revealed absent sensory nerve action potentials of sural nerve bilaterally, with slight decrease of the amplitude of the compound motor action potentials in the common peroneal and tibial nerves. Needle EMG was unremarkable. Somatosensory evoked potentials were compatible with a dysfunction of the posterior cord below cervical level. The clinical setting and electrophysiological studies were compatible with the diagnosis of a sensory ganglionopathy.

**Figure 2:**
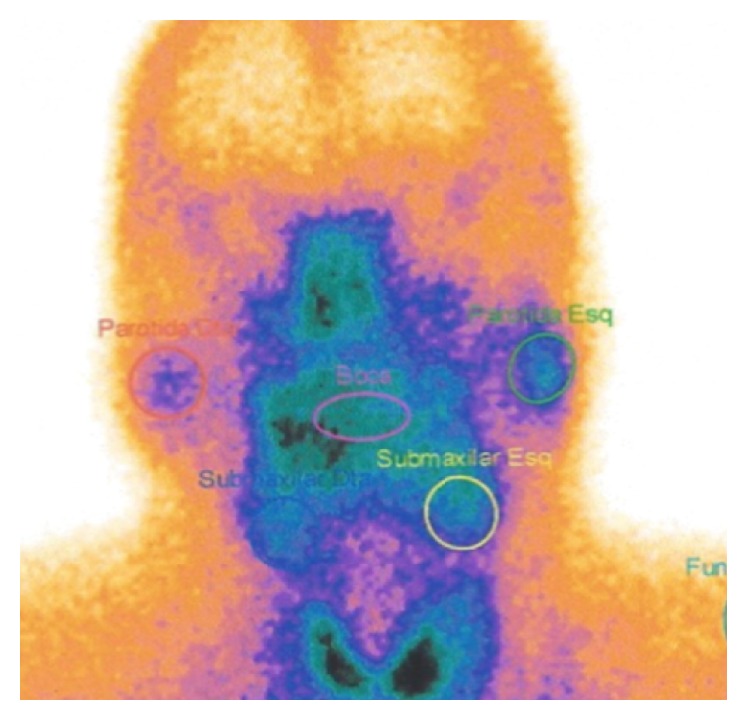
Salivary gland scintigraphy showing decreased uptake in submaxillary and parotid.

**Figure 3:**
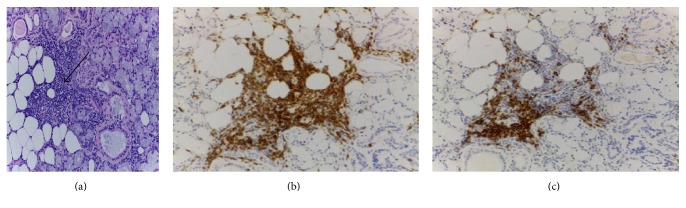
Submaxillary gland biopsy. (a) H&E 10 × 20—one focus of lymphocytic infiltrate (black arrow). (b) Immunohistochemistry—T lymphocytes (CD3+). (c) Immunohistochemistry—B lymphocytes (CD20+).

With this clinical scenario and steroid responsiveness, an immune-mediated disease was suspected. The complaints of xerostomia along with the parotiditis were suggestive of Sjögren syndrome. Anti-Ro (SSA) and anti-La (SSB) antibodies were repeatedly negative, as were rheumatoid factor and antinuclear antibodies (ANA). A salivary gland scintigraphy (99TcO4-) was performed and it revealed severely decreased uptake in submaxillary and parotid glands (Figure 2). Schirmer's test showed severe reduction of tear production (2 mm bilaterally after 5 minutes) and submaxillary gland biopsy (Figure 3) identified a multifocal inflammatory lymphocytic infiltrate with >1 focus/4 mm^2^ (1 focus = 50 lymphocytes), thus favouring the diagnosis of Sjögren syndrome.

Steroid therapy resulted in global improvement of psychiatric and cognitive symptoms, as well as of extrapyramidal features and sensory ataxia allowing for unassisted gait. Two months after admission there was a relapse of psychotic symptoms (delusions—the patient strongly believed that her son was in great danger; visual and tactile hallucinations—she saw bizarre animals and felt them touching her legs) that were refractory to oral steroids and antipsychotic drugs, which led to treatment escalation with cyclophosphamide. After 2 cycles of cyclophosphamide completed, recurrent urinary tract infections forced its withdrawal, with subsequent worsening of central and peripheral neurologic symptoms. Rituximab treatment was then initiated, in a regimen of two 1000 mg IV repeated twice with six months apart. Following this treatment, a progressive clinical improvement was observed.

One year after admittance, neurological reassessment showed that the patient was oriented in time and space, with attention and short- and long-term memory preserved. Cranial nerve examination was unremarkable. The superior limbs showed a dystonic posture of the wrist with athetosis of the fingers that were enhanced with the eyes closed. 

**Figure 4:**
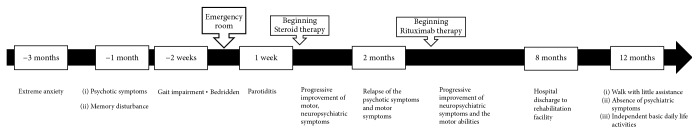
Diagram representing the timeline of the relevant clinical events. The time on the arrow refers to the date of emergency room admittance.

*Normal Strength and Muscle Tone*. Absent deep tendon reflexes, decreased vibration, and position senses on the four limbs, with sensory ataxia and Romberg sign, were observed. Gait was wide-based but possible with little assistance. The patient had no psychiatric symptoms and was independent in all basic daily life activities (Figure 4).

## 3. Discussion

In primary Sjögren syndrome, neurological manifestations can be protean and involve both peripheral and central nervous systems, as exemplified by our patient, whose clinical picture included cognitive impairment, hallucinations, extrapyramidal features, and sensory ataxia. To the best of our knowledge, such severe, simultaneous, and rapidly progressive symptoms have not been previously reported.

The exact pathophysiology of CNS clinical features remains unclear. It is thought to be related to an inflammatory process, affecting small vessels, without causing visible structural damage [2]. Some authors suggest other mechanisms such as direct infiltration of the CNS by mononuclear cells [1]. The term Nonvasculitic Autoimmune Meningoencephalitis was proposed by Caselli et al. in 1999 when describing 5 patients presenting with progressive cognitive decline, with both serologic evidence of autoimmunity and of tissue inflammation, benefiting from immunosuppression. Interestingly, two out of the five patients from this original report later received the diagnosis of Sjögren syndrome [3].

There is no clear topographical relationship between the clinical features and the imaging studies; hence brain MRI is not useful for diagnosis, showing abnormalities in only 30% of cases [4]. In this particular case no functional imaging was performed, so no clinical-anatomical correlation was done.

Regarding the peripheral nervous system involvement, there is no certainty about the mechanism either. Sensory ganglionopathy appears to be mediated by direct mononuclear infiltration and destruction of the dorsal root ganglion and the posterior columns of the spinal cord, promoted by the leaky basement membrane of the capillaries supplying these structures [1].

Since 1965 several sets of criteria were proposed for classification of Sjögren syndrome. The most widely accepted are the American-European Consensus Group (AECG) criteria (from 1996, later revised in 2002), with no significant sensitivity and specificity difference to the criteria recently issued by the American college of rheumatology (2012) [5]. Our patient meets Sjögren syndrome criteria according to AECG. SS-A and SS-B antibodies were negative, but their low sensitivity (40%) when nervous system is involved has been previously reported [4].

There is scarce evidence in the literature about Sjögren syndrome treatment. Data regarding efficacy of corticosteroids and other immunosuppressive agents in extraglandular involvement derive from studies designed for* sicca* symptoms. There is some evidence that Rituximab can be used as a rescue therapy in refractory extraglandular features [6].

## 4. Conclusions

- Systemic autoimmune disturbances can affect both central and peripheral nervous system and they can present subacutely.
- SS-A and SS-B antibodies are less sensitive in Sjögren syndrome when nervous system is involved.
- When neurological features occur in Sjögren syndrome they frequently precede* sicca* symptoms.
- Before the clinical picture of a rapidly progressive cognitive decline, a systemic and treatable cause must be ruled out. Once infectious cause is excluded, immunosuppression should be attempted, and a positive response should motivate an obstinate search for a specific immunomediated disease [7].
